# Assessment of the Influence Systemic Cryotherapy Exerts on Chosen Skin Scores of Patients with Atopic Dermatitis: Pilot Study

**DOI:** 10.1155/2020/5279642

**Published:** 2020-09-07

**Authors:** Magdalena Kepinska-Szyszkowska, Anna Misiorek, Monika Kapinska-Mrowiecka, Jan Tabak, Karolina Malina

**Affiliations:** ^1^Institute of Applied Sciences, University School of Physical Education in Krakow, Poland; ^2^Dermatology Ward, Stefan Żeromski Hospital in Krakow, Poland; ^3^Malopolska Centre for Cryotherapy, Al. Pokoju 82 Krakow, Poland

## Abstract

**Background:**

One of the most important tasks in the treatment of atopic dermatitis (AD) is alleviation of racking skin dryness and persistent pruritus, because these factors exert a significant influence on worsening patients' quality of life. Cryotherapy being a new form of rehabilitation in AD may supplement and support a long-term process of AD treatment, because it has anti-inflammatory and antipruritic effects and exerts a positive influence on the nervous system.

**Methods:**

14 adults (mean age 32 ± 10.8) with mild to moderate AD were enrolled. WBC (15 treatments in total) took place in winter 2018/2019. Patient skin parameters (hydration of the epidermis, sebum level, and skin pH level) were measured with probes produced by Courage + Khazaka Electronic GmbH.

**Results:**

Changes were observed in the hydration level of the epidermis. The SCORAD index evaluating the AD intensity level also changed (decreased).

**Conclusion:**

Due to these properties, hypothesis has been put forward that WBC can be an effective, supporting method in the treatment of AD.

## 1. Introduction

A number of factors exert an influence on proper functioning of the skin barrier. These include the level of hydration and skin pH, quantity and composition of intracellular lipids, corneocyte cellular properties, and their cohesion in the cornified layer. Visibly rough and dry skin in individuals with AD reflects its abnormal picture also in morphology. Corneocyte cohesion increases, and their diameter decreases. The cycle of cell migration from the basal layer to the cornified layer is extended, and a general number of layers in the stratum corneum are increased [1]. Dry skin in atopic patients is associated with abnormal activity of the hydrolipid barrier. One of the reasons for its abnormal function is an increased transepidermal water loss (TEWL), as well as increased pruritus and inflammation [2]. In cases of exacerbated dermatitis, TEWL may increase even four times, whereas during remission even twice, as compared with healthy skin [3].

In atopic skin, there is also another distribution of lipids; they are packed hexagonally. Moreover, the level of sphingosine (a component of many lipids) in an undamaged and damaged stratum corneum in patients with AD is significantly decreased as compared with that in healthy patients from a control group [4]. Skin pH also plays a role in proper functioning of the epidermal barrier. Proper pH of the skin surface and its deeper layer conditions maintain proper physiological flora and antimicrobial immunity of the skin. Proper skin pH should be slightly acidic. In women, it should be 4.5-5.9, whereas in men, it should be a little lower, i.e., 4.3-5.9. Studies have proven that skin pH increases in individuals with AD [1, 5, 6].

One of the most important tasks in the treatment of AD is alleviation of racking skin dryness and persistent pruritus, because these factors exert a significant influence on worsening patients' quality of life. Topical therapy of AD includes proper skin care, avoiding or limiting factors that exacerbate the symptoms, and, in cases of superinfections, proper antibiotic therapy and use of preparations containing calcineurin inhibitors or glucocorticosteroids [7].

Whole-body cryotherapy (WBC) relies on a short stay (2-3 minutes) in a special cryochamber at very low ambient temperature. These treatments have a positive effect on the endocrine, nervous, and immune systems. They also accelerate tissue regeneration, relieve pain, and suppress inflammation after intense physical effort [8]. Cryotherapy being a new form of rehabilitation in AD may supplement and support a long-term process of AD treatment, because it also has anti-inflammatory and antipruritic effects. Cryotherapy does not show any adverse reactions, and that is why it is a safe method for treating patients. As compared with phototherapy, it may be combined with other treatment methods, e.g., cyclosporin or tacrolimus. These treatments do not carry the risk for developing skin cancers, which could be side effects while using phototherapy [9]. The aim of the study was to evaluate changes in selected skin parameters after WBC in patients with AD.

## 2. Materials and Methods

### 2.1. Characteristics of Patients

The subjects were adults, who live in a big city and suffer from AD for a long time. Most of them had exacerbations of the disease every few months, and the severity of AD showed annual seasonality. The following inclusion criteria were used in the study: age above 18 years, no contraindications to WBC procedures, and clinically diagnosed with AD (with mild to moderate AD). Exclusion criteria of the research were as follows: lack of informed consent for research; patients with AD treated with phototherapy, cyclosporine A, and oral corticosteroids, which are topical calcineurin inhibitors such as pimecrolimus and tacrolimus; patients during or after immunotherapy; children and adolescents under 18 years of age; breastfeeding mothers and pregnant women; and patients with inflammatory, infectious, and autoimmune diseases and cancer. On the basis of a questionnaire completed by patients in the presence of a dermatologist, an analysis of AD was made. The questionnaire included basic information such as disease duration, symptoms, treatment, diagnostics for allergies, and family history. The next stage involved assessment of the level of AD advancements on the basis of the Scoring Atopic Dermatitis (SCORAD) index. Also, major and minor AD criteria presented by Hanifin and Rajka were taken into consideration [10].

Fourteen adults (mean age 32 ± 10.8, 7 men and 7 women) with mild to moderate AD were enrolled. The study was carried out in accordance with the Declaration of Helsinki. The methodology of the scientific project was approved by the Bioethics Committee at the District Medical Chamber in Krakow, Poland (opinion no. 239/KBL/OIL/2018). The study was registered at ClinicalTrials.gov under the number NCT03761199.

### 2.2. Measurement of Skin Parameters

Patient skin parameters were measured with probes produced by Courage + Khazaka Electronic GmbH. The level of hydration of the epidermis was examined with Corneometer® CM 825; Sebumeter® SM 815 was used to determine sebum levels of the skin, and Skin-pH-Meter PH 905 was applied to determine the skin pH level. There were 4 measurements of skin parameters performed in the following way: prior to the first cryotherapy treatment, directly after the first treatment, after the 15^th^ treatment, and after 3 weeks from the moment when the therapy was discontinued. The measurement was performed on the involved skin on the patient's hand (dorsal side) and on areas free from lesions on the forearm (inner side). A SCORAD index was used during the series of treatments to assess the changes in intensity levels of the skin lesions. Measurements were taken before the treatments, after the 15^th^ treatments, and 3 weeks from the day when examination came to an end.

### 2.3. Whole-Body Cryotherapy Procedures

Whole-body cryotherapy (15 treatments in total, once a day) took place at the Malopolska Centre for Cryotherapy, Al. Pokoju 82, Krakow, Poland. Having been consulted with a physician to exclude contraindications for WBC, patients were referred for treatments. Patients entered the cryogenic chamber wearing special shoes, thick shocks, shorts, gloves, and a headwear. In order to get the body used to low temperatures, patients spend 1.5 minutes in the main chamber on the first day. The next day, it was 2 minutes and then 3 minutes till the end of the therapy. WBC took place in winter 2018/2019. Patients did not use local anti-inflammatory preparations and systemic antihistamine drugs a week before the therapy and during the course of the study.

**Figure 1:**
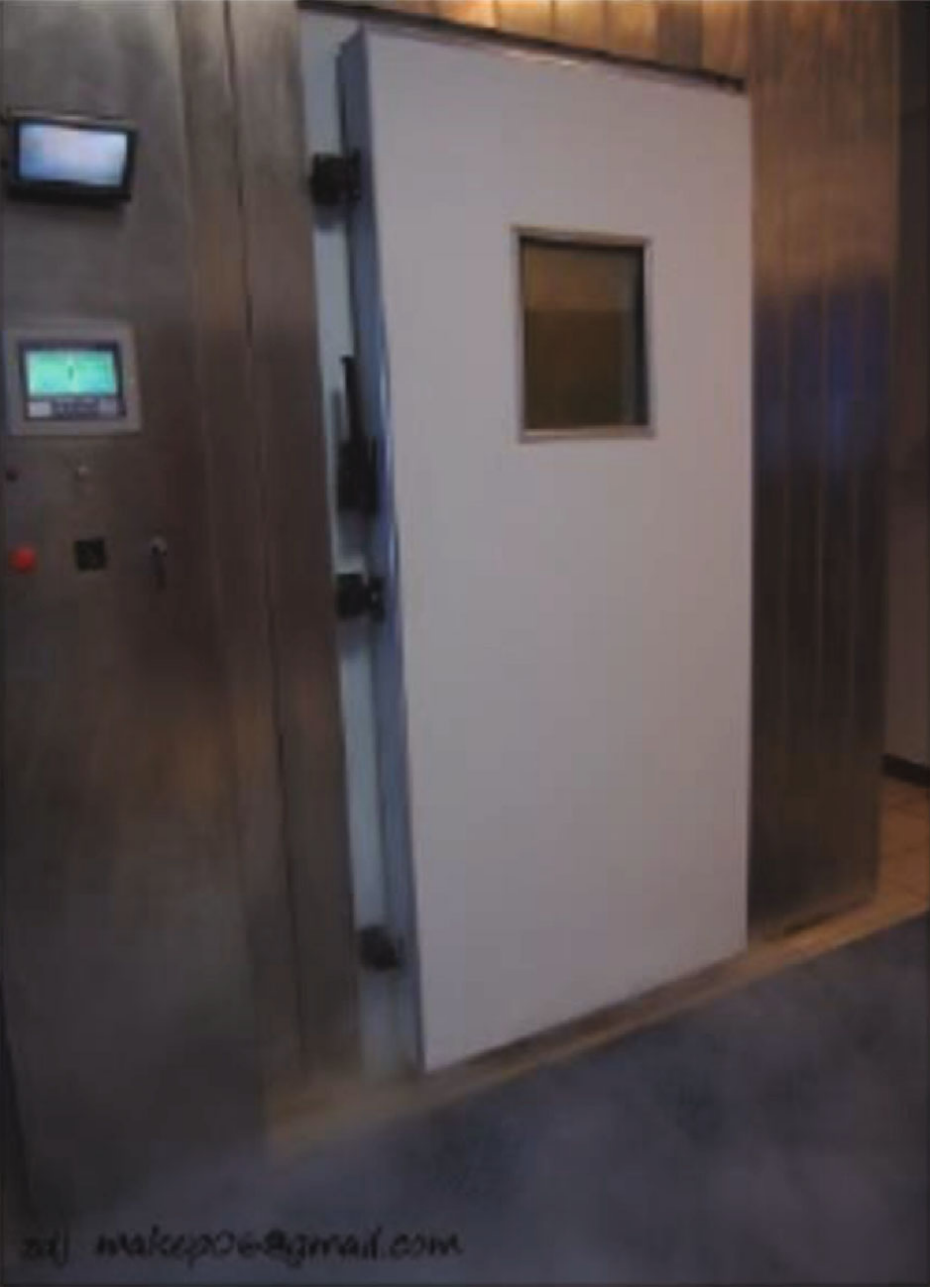
Entrance to the cryochamber (author's photograph).

**Figure 2:**
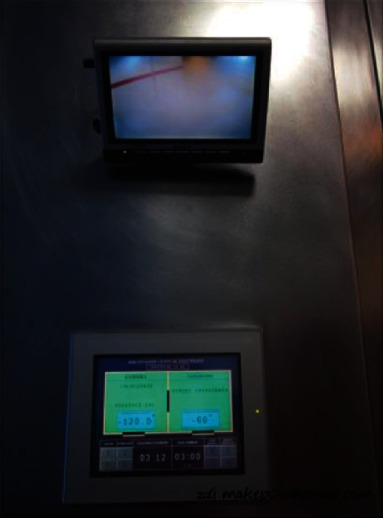
Camera and monitor indicating temperature inside the chamber (author's photograph).

Participants started to cool the body down in the vestibule in which the temperature was -60°C (30 seconds). Then, they proceeded to the main chamber, where the temperature was -120°C. Patients remained under continual control by a person handling the device thanks to thermal glass and intrachamber monitoring. Thanks to a phonic contact, it was possible to inform patients about the time remaining till the end of the treatment. In case of feeling unwell, patients could use an alarm button as well as an exit devise designed for opening the door from the inside (Figures 1 and 2).

### 2.4. Statistical Analysis

Due to a small number of participants and because of the lack of normal data distribution, nonparametric statistics (Wilcoxon signed-rank test) was used for comparisons of posttreatment data with the baseline. Median (*M*) and the value of the lower and the upper quartile (Q25-Q75) are reported in the subsequent sections and throughout the tables. A *p* value of less than 0.05 was considered statistically significant. All statistical analyses were performed with the Statistica 13 software (StatSoft, Inc., USA).

## 3. Results

Eight of fourteen patients completed the treatment period (5 women and 3 men). Of the patients who left the study, 2 patients discontinued owing to worsening dermatitis and the other 4 for reasons not related to skin condition. There were almost no differences in measurements of sebum skin levels in the measured areas of both the involved skin and skin without lesions. An exception is a measurement of the skin without atopic lesions, whose sebum level decreased; the result was statistically significant in comparison to measurement 4 to measurement 1 (*p* = 0.043). Changes in the level of hydration of the epidermis of both healthy skin and the skin with AD symptoms were also observed. In places where atopy symptoms occurred, the skin was drier as compared with the healthy area (Table 1). Furthermore, the results indicate that hydration of the epidermis in the involved skin increased directly after the 1^st^ treatment and after 3 weeks from the last treatment (2 vs. 1 (*p* = 0.015) and 4 vs. 1 (*p* = 0.010), respectively) (Table 2).

**Figure 3:**
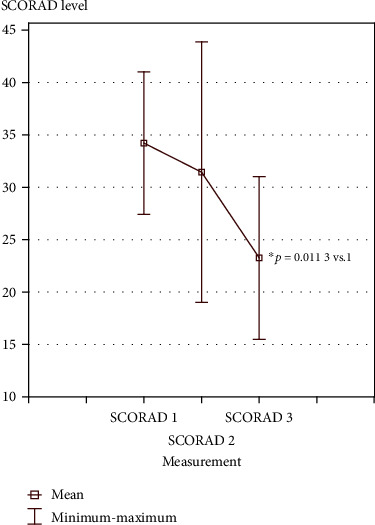
SCORAD index before the first cryotherapy treatment (1), after the 15th treatment (2), and 3 weeks after the last cryotherapy treatment (3**).**

There were no statistically significant differences in the pH level, both in the area of atopic lesions and in healthy skin. The SCORAD index evaluating the AD intensity level changed. The difference between the first (36.5 points) and the last (25.23 points) score was statistically significant (*p* = 0.011) (Figure 3).

## 4. Discussion

Applying a series of WBC in the treatment of AD is a new and unconventional solution. Studies of Klimenko et al. [9] are the only ones that evaluate the influence of WBC on the condition of patients with mild and moderate AD. A comparison of our studies with those mentioned above seems to be significant for this paper since it constitutes a reference to a larger group of patients. The patients used neither local anti-inflammatory preparations nor systemic antihistamine drugs a week before the therapy commencement and also during the course of the studies. Treatments in the cryochamber took place 3 times a week for a month. The level of transepidermal water loss (TEWL) was measured with VapoMeter. The mean TEWL value improved from 58.8 g/m^2^/h to 47.4 g/m^2^/h, which means better hydration of the epidermis [9]. In our studies, there were similar positive results, epidermal hydration increased directly after the first treatments in the involved skin, and also this effect was seen in 3 weeks after the end of the treatment. However, when comparing skin results with atopic changes to healthy skin results, the skin with atopic lesions was still very dry after cryotherapy treatments. According to studies by Knor et al. [1], skin with atopic lesions has a lower level of hydration than healthy skin of AD patients.

On the basis of an analysis of the collected data, it may be concluded that within the measurement area of the skin with lesions and uninvolved skin, almost no differences in sebum levels of the skin were noted. An exception is the measurement of the skin without atopic lesions whose sebum dropped in comparison to measurement 4 (3 weeks after the last WBC) to measurement 1 (before the first treatment of WBC) which means that the skin was very dry. Skin sebum levels in the patients, as it is presented above, did not improve after the treatments involving WBC. In line with epidemiological data, skin lesions caused by irritation appear more often in winter than in other seasons. Cold and dry air contributes to skin dryness and damage of the epidermal barrier [11]. It might be the reason behind the lack of improvement in skin sebum levels in our studies.

It was shown that there appears an increase in skin pH in patients with AD. A study conducted by Knor et al. [1] showed a higher pH concentration in individuals with atopic eczema as compared with individuals with perilesional and healthy skin and the control group [1]. Our studies also showed a slightly higher value in the pH on the hand with atopic eczema to compare with the forearm without lesions before the first treatment of WBC. In our studies, no changes in skin pH values (in both measured areas) were found after all WBC treatments and after 3 weeks from the last treatment. Skrzek et al. [12] also confirm the lack of influence of a series of WBC on the pH level in healthy people. It can be concluded that cryotherapy does not affect the skin pH [12].

In the presented results, a mean SCORAD index decreased after cryotherapy treatments and 3 weeks after the last treatment. Klimenko et al. [9] also observed decrease in the SCORAD index after a series of WBC treatments. According to the authors, cryotherapy of the entire body shows a steroid effect with additional statistically and clinically significant improvements in the case of pruritus and sleep [9]. Improvements in lesions in the patients (SCORAD index) might have also been influenced by good mood that accompanies people regularly using treatments in a cryochamber. When stressed, AD patients often experience pruritus. The neuroendocrine system and the immune system are connected to one another, and moreover, there are interactions between these systems and the skin. Stress exerts a negative influence on dermatoses, especially the inflammatory ones, such as AD [13]. Rymaszewska et al. [14] in their studies confirmed the efficacy of WBC for patients suffering from episodes of depression and anxiety. There was an improvement in the well-being and quality of life of the patients [14].

The beneficial changes after WBC treatments observed in our studies (changes in hydration of the epidermis and changes in the SCORAD index) may also result from the anti-inflammatory effect and from the effect on oxidative stress of WBC. Research shows that cryotherapy improves the body's antioxidant capacity and can be used as an adjunctive therapy in the treatment of diseases with oxidative stress [15]. According to Stanek et al. [16] after a series of 10 WBC treatments, the level of C-reactive protein and mucoproteins decreased in patients with ankylosing spondylitis (AS). In turn, other studies by Stanek et al. [17] also in the group of patients with AS additionally observed a decrease in markers of oxidative stress and changes in the activity of some antioxidative enzymes and in nonenzymatic antioxidant parameters. However, in the study of Banfi et al. [18] after a short-term WBC session in athletes, a decrease in creatine kinase and lactate dehydrogenase was observed along with a decrease in the inflammatory cytokine and an increase in the anti-inflammatory cytokine. Pournot et al. [19] explain the anti-inflammatory effect of vasoconstriction at the muscular level and the decrease in the inflammatory cytokine activity. The antioxidant activity of WBC treatments is also confirmed by Stanek et al. [20]. In the subjects (healthy men) studied, a decrease in the concentration of most parameters of oxidative stress was accompanied by an increase in the concentration of nonenzymatic antioxidants (total status of antioxidants and uric acid). Similar changes were observed by Lubkowska et al. [21]. Repeated WBC treatments cause changes in the state of peroxides and antioxidants, and these changes may depend on the number of treatments. WBC treatments also have an analgesic effect [22, 23], which could improve the skin condition of the subjects in our studies and contribute to lowering the SCORAD index.

The present study has some limitations. The study should involve a larger number of AD patients, both with and without WBC treatments.

## 5. General Conclusion

Based on our own research, WBC can be an effective, supporting method in the treatment of AD. However, there is still little data in the scientific literature on the effect of cryogenic temperatures on skin parameters in patients with AD.

## Figures and Tables

**Table 1:** The skin hydration measured before the first treatment (1), immediately after the first (2), after the 15th treatment (3), and after 3 weeks from the end of WBC (4) on healthy and diseased skin.

Measurement no.	Hydration level		*p*
	Forearm without atopic changes (F) *M* (Q25-Q75)	Hand with atopic changes (H) *M* (Q25-Q75)	
1	29.9 (28.1-32.3)	10 (8-12.1)	1F vs. 1H 0.011 1F vs. 2H 0.011 1Fvs.3H 0.011 1F vs. 4H 0.011

2	28.1 (22.4-29.4)	10.5 (9.4-13.5)	2F vs. 1H 0.011 2F vs. 2H 0.025 2F vs. 3H 0.011 2F vs. 4H 0.011

3	28.2 (25.8-30.3)	9.9 (7.9-16.4)	3F vs. 1H 0.017 3F vs. 2H 0.011 3F vs. 3H 0.011 3F vs. 4H NS

4	23.5 (14.5-30.1)	14.8 (9.5-20.0)	4F vs. 1H 0.011 4F vs. 2H 0.035 4F vs. 3H 0.011 4F vs. 4H NS

1-4: measurement number; vs.: versus; NS: nonstatistical; F: forearm; H: hand; *M*: median; Q25-Q75: the value of the lower and the upper quartile. *p* < 0.05.

**Table 2:** The skin parameters measured before the first treatment (1), immediately after the first (2), after the 15th treatment (3), and after 3 weeks from the end of WBC therapy (4) on healthy and diseased skin.

Part of the body	Measurement				*p*
	1	2	3	4	
Forearm without atopic changes	Hydration level *M* (Q25-Q75)				
	29.9 (28.1-32.3)	28.1 (22.4-29.4)	28.2 (25.8-30.3)	23.5 (14.5-30.1)	NS
	Sebum level *M* (Q25-Q75)				
	1 (0-1)	1 (1-2)	0.5 (0-1)	0 (0-0)	4 vs.1 0.043
	pH level *M* (Q25-Q75)				
	5.6 (5.2-6.1)	5.4 (5.1-5.9)	5.4(4.5-6.0)	5.7 (4.8-6.0)	NS

Hand with atopic changes	Hydration level *M* (Q25-Q75)				
	10 (8-12.1)	10.5 (9.4-13.5)	9.9 (7.9-16.4)	14.8 (9.5-20.0)	2 vs.1 0.015
					3 vs.1 0.010
					4 vs.1 0.010
	Sebum level *M* (Q25-Q75)				
	1 (0-1)	1 (0-2)	0 (0-3)	1 (0-1)	NS
	pH level *M* (Q25-Q75)				
	5.8 (5.5-5.9)	5.6 (5.6-6.0)	5.5 (5.0-6.08)	5.6 (5.3-5.8)	NS

1-4: measurement number; vs.: versus; NS: nonstatistical; *M*: median; Q25-Q75: the value of the lower and the upper quartile. *p* < 0.05.

## Data Availability

The data used to support the findings of this study are included within the article.
